# The first phytochemical report of *Galanthus transcaucasicus* Fomin

**Published:** 2010

**Authors:** M.H. Salehi Sourmaghi, B. Azadi, Gh. Amin, M. Amini, M. Sharifzadeh

**Affiliations:** 1 Department of Pharmacognosy; 2 Department of Medicinal Chemistry; 3 Department of Toxicology and Pharmacology, Faculty of Pharmacy and Research Center of Medicinal Plants, Tehran University of Medical Sciences, Tehran, Iran

**Keywords:** *Galanthus transcaucasicus*, Galanthamine, Lycorine, Tazettine, Isoquinoline alkaloids

## Abstract

**Background and the purpose of study:**

*Galanthus transcaucasicus* Fomin (Amaryllidaceae) is an endemic species to the Caucasia and Alborz mountains in Iran which locally named “Gole-Barfi”. While there are many reports on pharmacological activities of *Galanthus* species’ alkaloids, there is no report on *G. transcaucasicus* and this article is the first phytochemical study on this species.

**Methods:**

Extracted alkaloids from *G. transcaucasicus* bulbs were isolated using different chromatographic methods and the structures of the components were determined by physical and spectroscopic data.

**Results:**

Five isoquinoline type alkaloids namely galanthamine (8.04%), narwedine (6.90%), lycorine (19.48%), caranine (3.45%) and tazettine (5.75%) of total alkaloid extract were isolated from the bulbs of *Galanthus transcaucasicus* Fomin.

**Major conclusion:**

Because of the presence of biologically active alkaloids especially galanthamine and the major alkaloid lycorine in Gol-e-Barfi, the plant may be used as a natural source for pharmaceutical purposes.

## INTRODUCTION

*Galanthus* is an important genus of the Amaryllidaceae family and the species are native to many parts of Europe including Bulgaria, the eastern parts of Turkey, the Caucasus Mountain and Iran (1, 2). They are bulbous plants with narrow grassy leaves, erect flowering stalks and white flowers (3). The name *Galanthus* is derived from two Greek words “gala” for milk and “anthos” for flower, which are descriptive of the snow white blossoms of this genus (4). The majority of alkaloids especially isoquinoline type like as galanthamine, lycorine, caranine, narciclasine, tazettine, narwedine and montanine which have a wide range of acetylcholinesterase inhibitory, antitumor, antiviral, immunostimulatory and antimalarial activities, have been isolated from this genus species (5). *Galanthus transcaucasicus* Fomin (snowdrop) which is locally named “Gol-e-Barfi “is an endemic species of the Caucasia and the Alborz mountains in Iran (2, 6). A thorough literature survey revealed that there is no report on this species and this article describes, identification of alkaloids especially galanthamine and lycorine from *Galanthus transcaucasicus* Fomin.

## MATERIALS AND METHODS

### 

#### General

Melting points were determined on a Reichert-Jung apparatus and are uncorrected. FT-IR spectra were recorded using a Nicolet 550-A spectrometer (KBr disks). EIMS spectra were recorded by a Hewlett Packard 5973 Mass Spectrometer at 70 eV. NMR spectra were acquired at 500 MHz for ^1^H and 125 MHz for ^13^C, on a Bruker Avance 500 spectrometer, using CDCl3 and CD_3_OD as solvents and TMS as internal standard. Chemical shifts are expressed in ▵ (ppm) and coupling constants (J) in Hz. TLC aluminium sheets (silica gel 60 F_254_ 20 × 20), silica gel 60GF_254_ and silica gel (70-230 mesh) were used for analytical and preparative TLC and column chromatography, respectively. Sephadex LH-20 was used for gel filtration. With exception of Sephadex LH-20 and galanthamine standard which were from Pharmacia and Sigma-Aldrich companies, respectively, all other chemicals were provided by Merck company.

Spots on chromatograms were detected under UV light (254 and 366 nm) and Dragendorff's reagent.

#### Plant material

*Galanthus transcaucasicus* Fomin specimens were collected in April 2008 from the west (Rostam Abad of Rood Bar) and east (Sang Deh of Sari) of Alborz Mountain in Iran. Voucher specimens were identified by Dr.Gholamreza Amin and are deposited under No. 6670-TEH and No. 6711-TEH, at the herbarium of Faculty of pharmacy, Tehran University of Medical Sciences, Tehran, Iran.

#### Extraction and isolation

The air-dried and powdered bulbs of *Galanthus transcaucasicus* from the west and east of Alborz mountains (2 and 1 kg respectively) were extracted by percolation method with 96° EtOH (17.6 and 12.1 liter. respectively) at room temperature. The ethanol extracts were evaporated under reduced pressure to give brown gummy extracts (236.2 and 57.5 g, yield: 11.81% and 5.75% respectively) (7).

The crude extracts were dissolved in 2% aq. HCl separately. The acidic solution was filtrated and basified to pH 9 -10 by 25% aq. NH_4_OH and then extracted successively with CHCl_3_. The organic solvents were dried over anhydrous Na_2_SO_4_ and evaporated to give the crude alkaloid extracts. The basic crud alkaloid extract was then fractionated by column chromatography on silica gel by gradient elution with petroleum ether (40-60°), petroleum ether- chloroform, chloroform, chloroform-methanol and finally, methanol. Forty and eight fractions (50 ml each) were collected and monitored by TLC using CHCl3-MeOH (7:3) and CHCl3-EtOAc-MeOH (3:4:3) as solvent systems. Fractions obtained by 5% to 80% MeOH-CHCl3 contained alkaloids. By mixing similar fractions 4 sub-fractions are obtained. Further purification was carried out by successive preparative TLC, preparative column chromatography and Sephadex LH-20. Since during this investigation it was found that the compounds are unstable, following prompt method was used for isolation and purification of alkaloids.

The crude alkaloid extract was dissolved in MeOH from which lycorine (III), the major alkaloid, crystallized directly (8). The solution was concentrated and subjected to preparative TLC using CHCl_3_-MeOH (7:3) as mobile phase to afford galanthamine (I) and caranine (IV) (*Rf*: 0.58 & 0.72). The spot with *Rf*:0.50–0.54 was subjected to further fractionation and purification by preparative TLC using CH_2_Cl_2_ -MeOH (7:1) as the solvent system to yield narwedine (II) (*Rf*: 0.44) and tazettine (V) (*Rf*: 0.33) (8). The compounds were separated from the layers with MeOH.

#### Galanthamine (I)

White needles, m.p. 124-126 °C. FT - IR *v*_max_ cm^-1^:3300, 1620, 1595, 1515, 1420, 1280, 1040. EIMS 70 eV, *m/z* (rel. int.): 287[M]^+^ (92), 286 (100), 244 (26), 230 (15), 226 (18), 216 (33), 174 (28). 1H-NMR (500MHz,CDCl_3_): δ 1.60 (1H, *dd, J* = 13.5, 2.3 Hz, H-11β), 2.03(1H,*ddd, J* = 15.7, 5.0, 2.5 Hz, H-2α), 2.11 (1H, *td, J* = 13.5, 3.2 Hz, H-11α), 2.42(3H, *s*, NMe), 2.71(1H, *dt, J*= 15.7, 1.8 Hz, H-2β), 3.08(1H, *br d, J* = 14.6 Hz, H-12α), 3.30(1H, *br t, J* = 14.6 Hz, H-12β), 3.71 (1H, *d, J* = 15.2 Hz, H-6α), 3.85(3H, *s*, OMe), 3.89(1H, *s,* 3-OH), 4.12(1H, *d, J* = 15.2 Hz, H-6β), 4.16(1H, *t, J* = 4.5 Hz, H-3),4.63(1H,*br s,* H-1), 6.02(1H, *dd, J* = 10.2, 4.9 Hz, H-4), 6.08(1H, *d, J* = 10.2 Hz, H-4a), 6.64(1H, *d, J* = 8.2 Hz, H-7), 6.68 (1H, *d, J =* 8.2 Hz, H-8) (9).

#### Narwedine (II)

White amorphous powder, m.p. 192-193°C. EIMS 70 eV, *m/z* (rel. int.): 285 [M^+^] (93), 284 (100), 242 (27), 216 (28), 199 (27), 174 (37). 1H-NMR (500 MHz, CD_3_OD and CDCl_3_): δ 2.14 – 2.24 (2H, m, H-11α and H-11β), 2.45 (3H, *s*, NMe), 2.72 (1H, *dd, J* = 17.5, 4.0 Hz, H-2α), 3.08 (1H, *dd, J* = 17.5, 1.5 Hz, H-2β), 3.14 – 3.27(2H, covered by solvent peak, H-12α and H-12β), 3.78 (3H, *s*, OMe), 3.81 (1H, *d, J* = 12.0 Hz, H-6), 4.20 (1H, *br d, J* = 12.0 Hz, H-6’), 4.69 (1H,*br s*, H-1), 6.00 (1H, *d, J* = 10.4 Hz, H-4), 6.64 (1H, *d, J* = 8.4 Hz, H-7), 6.67 (1H, *d, J* = 8.4 Hz, H-8), 6.87 (1H,*d,J* = 10.8 Hz, H-4a) (10).

#### Lycorine (III)

White needles, m.p. 270°C. FT-IR *v*max cm^-1^: 3324, 2865, 1503, 1486, 1356, 1312, 1262, 1238, 1038, 1001. EIMS 70 eV, *m/z* (rel. int.): 287 [M]^+^ (41), 286 (22), 268 (27), 250 (12), 228 (14), 227 (79), 226 (100), 147 (11). 1H-NMR (500MHz, CD_3_OD): δ 2.44 (1H, *dd, J* = 14.8, 9.0 Hz, H-12α), 2,56 – 2.73 ( 3H, *m,* H-11α, β and H-10b), 2.88 (1H, *d, J* = 10.5 Hz, H-4a), 3.35(1H, *dd, J* = 14.4, 7.5 Hz, H-12β), 3.55(1H, *dd, J* = 14.2, 1.2 Hz, H-6α), 4.13(1H, *d, J* = 14.2 Hz, H-6β), 4.18 (1H, *br, s,* H-2α), 4.48 (1H, *s,* H-1), 4.61 (2H, *br s*, 1-OH and 2-OH), 5.56 (1H, *br s,* H-3), 5.92 (2H, *s,* OCH_2_O), 6.65 (1H, *s,* H-7), 6.88 (1H, *s,* H-10). 13C-NMR (125 MHz, CD_3_OD): δ 29.3 (C-11), 41.4 (C-10b), 54.7 (C-12), 57.8 (C-6), 62.4 (C-4a), 71.9 (C-1), 73.2 (C-2), 102.3 (OCH_2_O), 106.0 (C-10), 108.2 (C-7), 119.1 (C-3), 129.8 (C-10a), 130.4 (C-6a), 143.8 (C-4), 147.7 (C-8), 148.3 (C-9) (11).

#### Caranine (IV)

White amorphous powder, m.p. 178-180°C. 1H-NMR (500MHz, CD3OD and CDCl_3_): ▵ 2.13 (1H, *t, J* = 18.6 Hz, H-12α), 2.28 (1H, *d, J* = 7.7 Hz, H-10b), 2,42-2.57 (3H, *m,* H-2α, β and H-11), 2.92 – 2.95 (1H, *m,* H-4a), 3.18-3.23 (1H, *m,* H-12β), 3.67 (1H, *dd, J* = 19.2, 3.5 Hz, H-6α), 3.96 (1H, *d, J* = 19.5 Hz, H-6β), 4.29 (1H, *br s,* 1-OH), 4.60-4.64 (1H, *m,* H-1), 5.41 – 5.64 (1H, *m,* H-3), 5.73 and 5.74 (2H, *2d, J* = 1.4 Hz, OCH_2_O), 6.43 (1H, *s,* H-7), 6.71 (1H, *s,* H-10). 13C-NMR (125 MHz, CD_3_OD and CDCl_3_): δ 28.8 (C-11), 32.2 (C-2), 39.4 (C-10b), 54.1 (C-12), 56.2 (C-6), 61.5 (C-4a), 71.9 (C-1), 101.5 (OCH_2_O), 105.4 (C-10), 107.7 (C-7), 119.4 C-3), 130.4 (C-10a), 130.9 (C-6a), 142.0 (C-4), 146.8 (C-8), 147.6 (C-9) (11).

#### Tazettine (V)

White crystalline powder, m.p. 195-198°C. FT-IR *v*_max_ cm^-1^: 3196, 1475, 1453, 1380, 1127, 883, 706. EIMS 70 eV, *m/z* (rel. int.): 331 [M+] (30), 316(15), 298 (22), 247 (100), 201 (15), 181 (12), 152 (10). ^1^H-NMR (500 MHz, CD_3_OD and CDCl_3_): δ 1.63 (1H, *ddd, J* = 15.8, 5.2, 2.6 Hz, H-4β), 2.23 (1H, *dm, J,* = 15.8 Hz, H-4α), 2.58 (3H, *s*, NMe), 2.71(1H, *d, J* = 10.8 Hz, H-12β), 2.83 – 2.94 (1H, *m*, H-4a), 3.17 (1H, *d, J*= 10.8 Hz, H-12α), 3.49 (3H, *s*, OMe), 3.84 (1H, *br s*, 11-OH), 4.06 – 4.20 (1H, *m*, H-3), 4.64 (1H, *d, J* = 15.1 Hz, H-6β), 4.97 (1H, *d, J* = 15.8 Hz, H-6α), 5.35 -5.39 (1H, *m,* H-1), 5.78 and 5.79 (2H, 2*s,* OCH_2_O), 5.92 – 5.97 (1H, *m,* H-2), 6.37 (1H, *s,* H-7), 6.72 (1H, *s,* H-10) (12).

## RESULTS AND DISCUSSION

**Figure 1:**
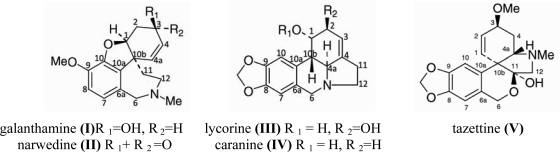
Structures of compo I-V.

The alkaloid content of the total extract of bulbs of *Galanthus transcaucasicus* Fomin from the east and west of Alborz Mountains were 5.50% and 4.27%, respectively and this variability may be justified by alkanity of the soil of the east compared to the west regions. A reported articles on the other species of *Galanthus* showed that the alkaloid contents in bulbs of *G. nivalis, G. gracilis* and *G. plicatus* growing in Turkey were 0.52%, 1.41% and 2.42% of total extracts (13–15) and for *G. nivalis* and *G. elwesii* growing in Bulgaria were 0.04% and 0.088% respectively (16, 17). The major alkaloids of the reported species were tazettine (38.3%) and 8-Odemethylhomolycorine (31.8%) (8, 16, 17). The basic chloroform extract of the bulbs of *Galanthus transcaucasicus* Fomin afforded the isoquinoline type alkaloids such as galanthamine (8.04%), narwedine (6.90%), lycorine (the major alkaloid 19.48%), caranine (3.45%) and tazettine (5.75%) (Fig. 1).

Galanthamine, the most important alkaloid, is a long acting, competitive and reversible acetylcholinesterase inhibitor for mid-to-moderate Alzheimer's disease (18). Narwedine, the biogenetic precursor of galanthamine, has been used as a respiratory stimulant (5). It inhibits the action of narcotics and hypnotics and enhances the analgesic activity of morphine as well as the pharmacological effects of caffeine, carbazole, arecoline and nicotine (19). Lycorine, has appreciable AChE inhibitory activity. It is an analgesic, like aspirin, and has hypotensive activity. Additionally, lycorine has antiviral, antitumor, antimalarial, anti-inflammatory, antiplatelet, emetic and cytotoxic activities (11). Caranine, another lycorine type alkaloid, is a hypotensive which also has shown AChE activity (11). Tazettine is mildly active against certain tumor cell lines, it also displays weak hypotensive and antimalarial activities and it has been reported as an extraction artifact from pretazettine (11).

On the basis of the total alkaloid content of *Galanthus transcaucasicus* Fomin especially its major alkaloid lycorine, this species will be subjected to further investigation.
